# Supplementary material for: Paeniclostridium sordellii hemorrhagic toxin targets TMPRSS2 to induce colonic epithelial lesions
Source: Nat Commun. 2022 Jul 26;13:4331. doi: 10.1038/s41467-022-31994-x (PMC9321280; doi:10.1038/s41467-022-31994-x)

# Supplementary Information

# *Paeniclostridium sordellii* hemorrhagic toxin targets TMPRSS2 to induce colonic epithelial lesions

Xingxing Li[1,2,3,4,#], Liuqing He[1,2,3,4,#], Jianhua Luo[2,3,4], Yangling Zheng[2,3,4], Yao Zhou[2,3,4], Danyang Li[2,3,4], Yuanyuan Zhang[2,3,4], Zhenrui Pan[2,3,4], Yanyan Li[2,3], and Liang Tao[1,2,3,4,*]

[1] Fudan University, Shanghai, 200433, China
[2] Key Laboratory of Structural Biology of Zhejiang Province, School of Life Sciences, Westlake University, Hangzhou, Zhejiang, 310024, China
[3] Center for Infectious Disease Research, Westlake Laboratory of Life Sciences and Biomedicine, Hangzhou, Zhejiang, 310024, China
[4] Institute of Basic Medical Sciences, Westlake Institute for Advanced Study, Hangzhou, Zhejiang, 310024, China

[#] These authors contributed equally to this work.
* Corresponding to:
Liang Tao, Ph.D. Email: taoliang@westlake.edu.cn

## Supplementary Figures

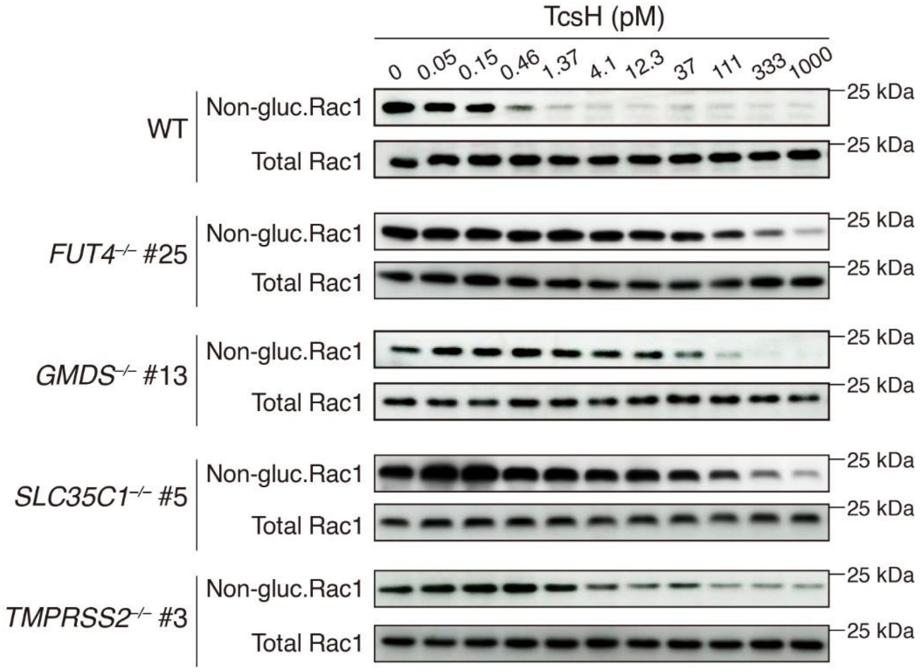


**Supplementary Fig. 1 | RAC1 glucosylation of MCF-7 WT and KO cells post-exposed to TcsH.** The sensitivities of MCF-7 WT and KO cells to TcsH were compared by analyzing the glucosylation level of RAC1 via immunoblot assays. The experiments have been repeated independently twice with similar results.

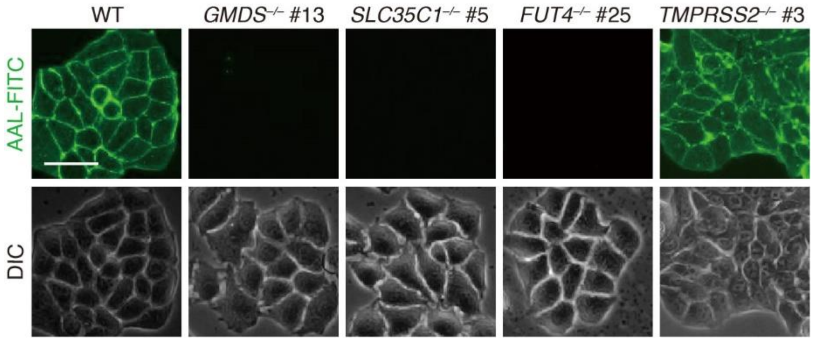


**Supplementary Fig. 2 | Cell surface binding of AAL-FITC to MCF-7 WT and KO cells.** Fluorescence microscopy showed that AAL-FITC strongly binds to the MCF-7 WT and *TMPRSS2*$^{-/-}$ cells, but not others. The scale bar represents 50 μm. The experiments have been repeated independently three times with similar results.

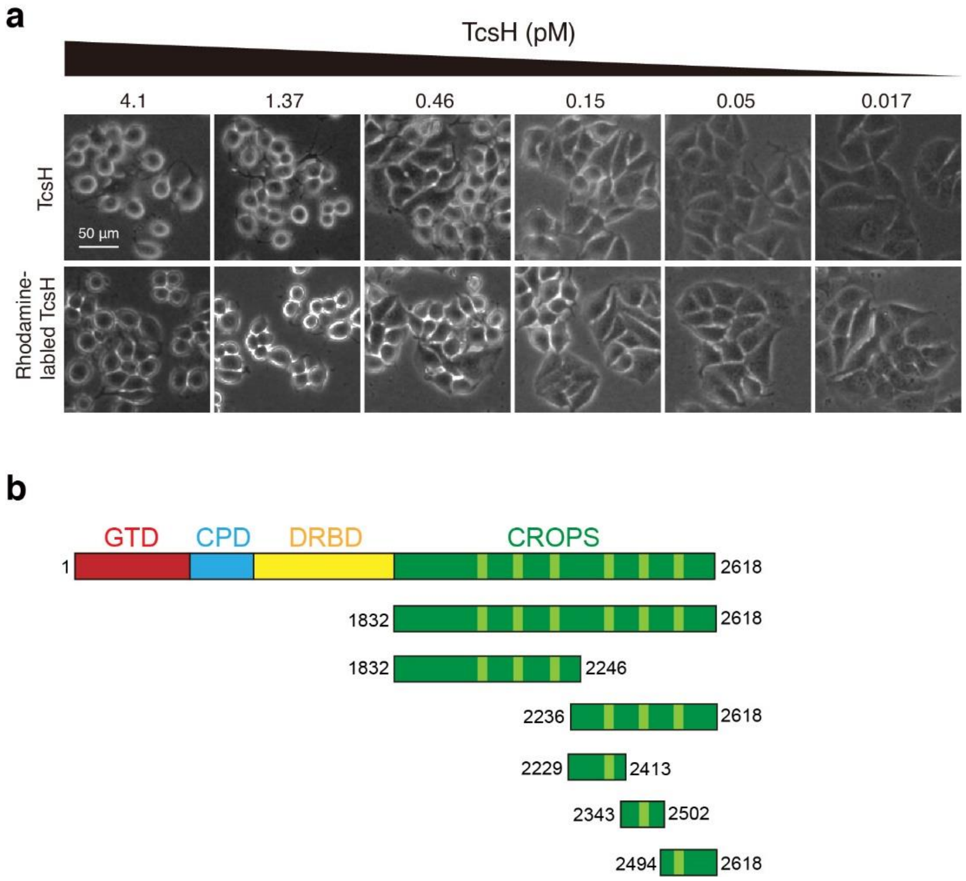


**Supplementary Fig. 3 | Rhodamine-labeling does not affect the TcsH activity**.

**a,** MCF-7 cells were incubated with either TcsH or Rhodamine-labeled TcsH of indicated concentrations for 12 h. Similar levels of toxin-induced cytopathic effects were observed. The scale bar represents 50 µm. The experiments have been repeated independently three times with similar results. **b,** Schematic illustrations of different TcsH CROPs fragments used in this study.

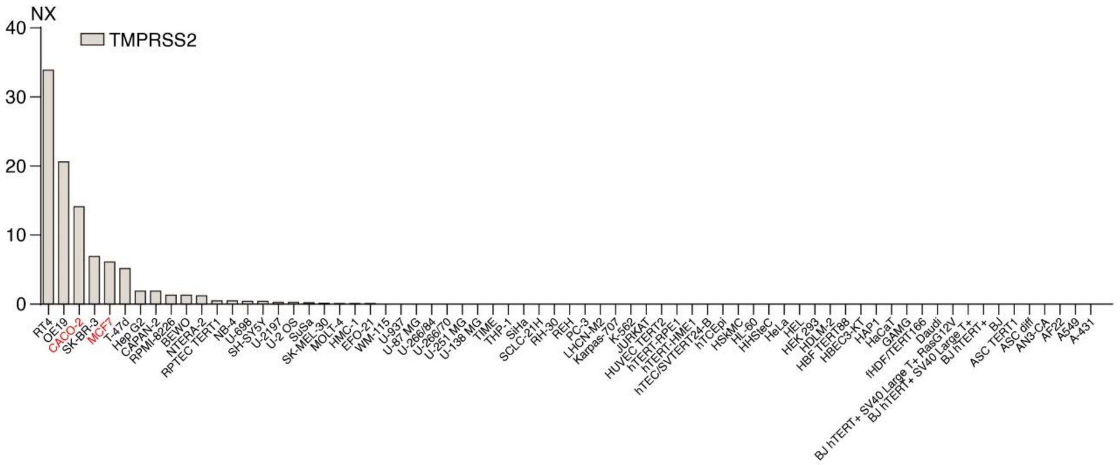


**Supplementary Fig. 4 | Expression levels of TMPRSS2 in various human cells.** Transcriptional levels of *TMPRSS2* in various human cell lines are based on the data obtained from the Human Protein Atlas (https://www.proteinatlas.org/). Cell lines marked by red were tested in this study.

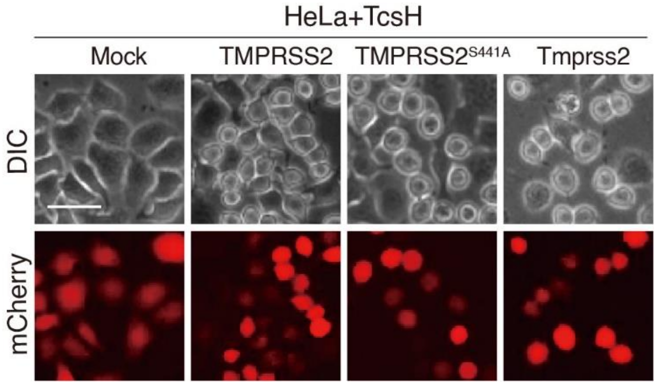


**Supplementary Fig. 5 | Ectopic expression of TMPRSS2 sensitizes HeLa cells to TcsH.** The HeLa cells were transfected with a human TMPRSS2, TMPRSS2$^{S441A}$, or mouse Tmprss2, followed by exposure to 2 nM TcsH for 3 hours. Representative images are shown. Red fluorescence (mCherry) marked transfected cells. The scale bar represents 50 μm. The experiments have been repeated independently three times with similar results.

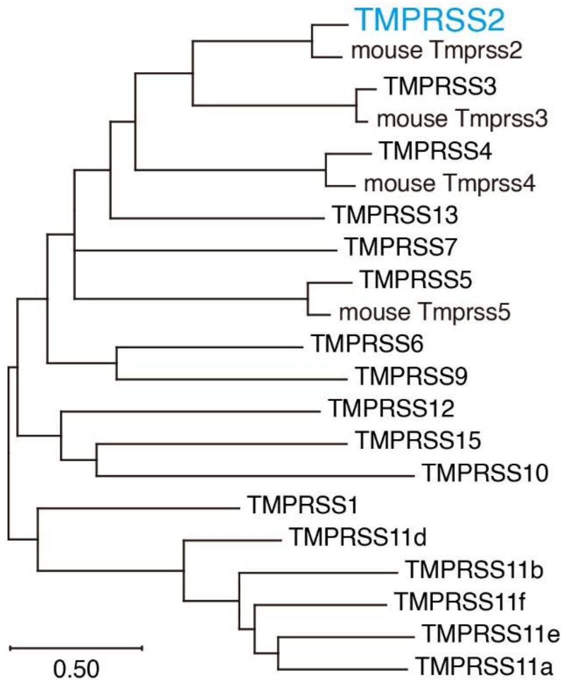


**Supplementary Fig. 6 | Phylogenetic analysis of TMPRSS subfamily proteins.** Phylogenetic tree of some human and mouse TMPRSS proteins generated by the Neighbor-joining method.

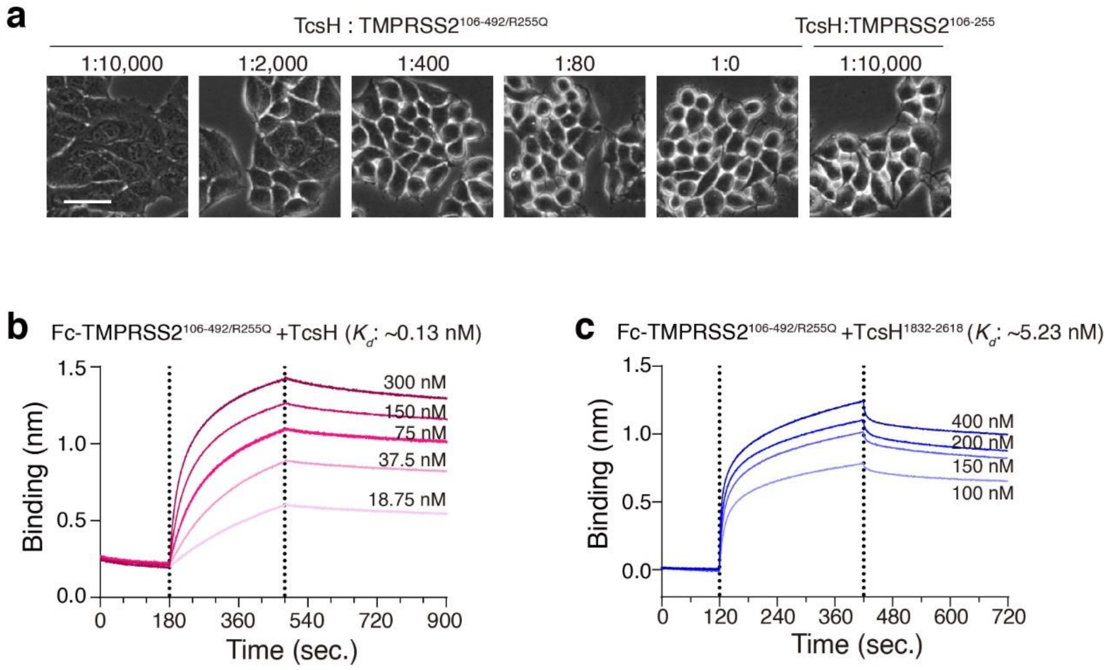


**Supplementary Fig. 7 | The ectodomain of TMPRSS2 directly binds to TcsH. a,** Fc-TMPRSS2$^{106\text{-}492/R255Q}$ protects the MCF-7 cells from TcsH (10 pM, 3.5 hours), measured by the cell-rounding assay. Representative images are from one of three independent experiments. The scale bar represents 50 µm. **b,** Representative binding curves of TcsH to Fc-TMPRSS2$^{106\text{-}492/R255Q}$. **c,** Representative binding curves of TcsH$^{1832\text{-}2618}$ to Fc-TMPRSS2$^{106\text{-}492/R255Q}$.

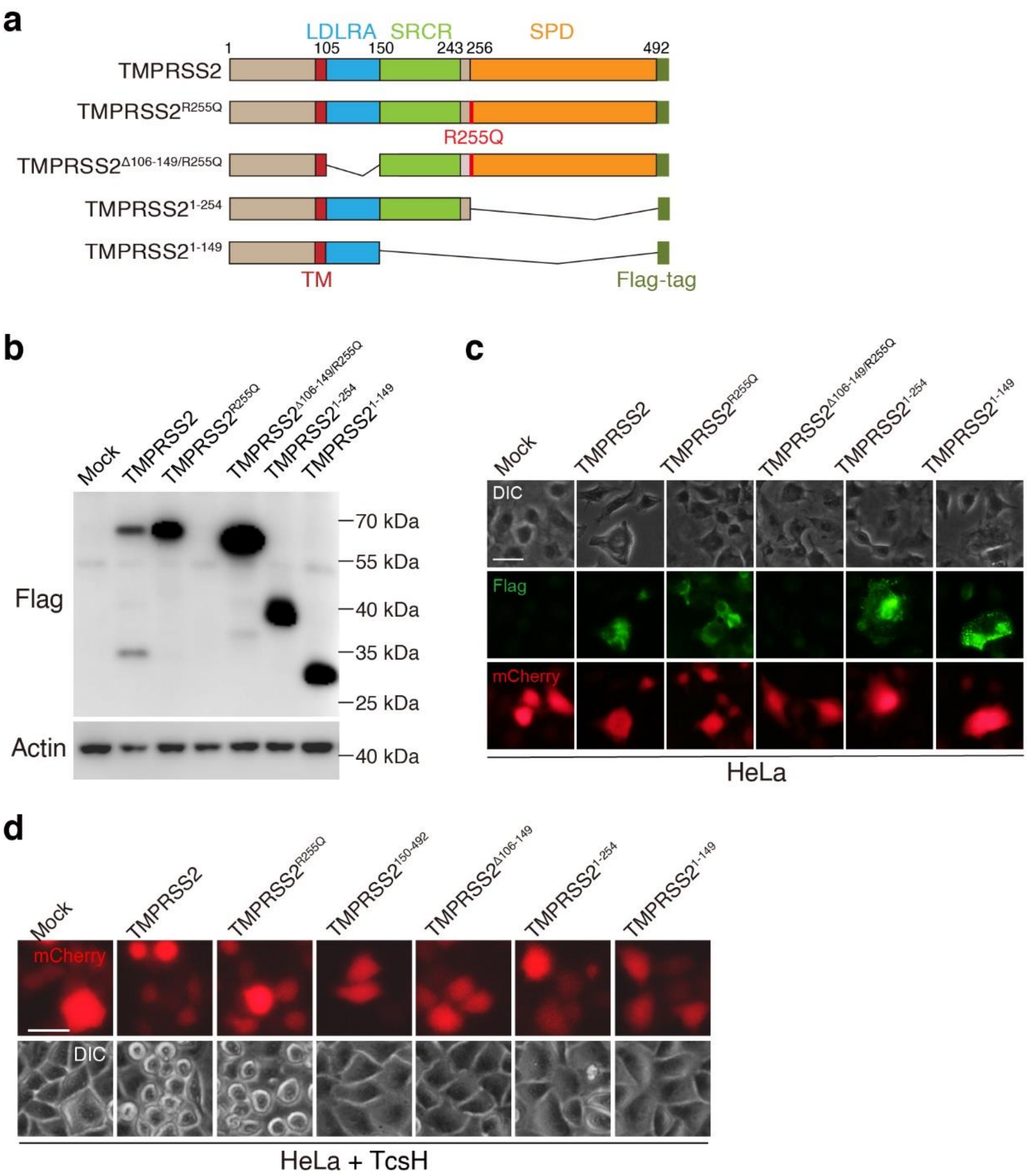


**Supplementary Fig. 8 | SPD of TMPRSS2 is important for mediating the TcsH entry. a,** Schematic illustrations of different TMPRSS2 constructs used in this study. **b,** Ectopic expression of the TMPRSS2 constructs in the HeLa cells was monitored by immunoblot. The experiments have been repeated independently twice with similar results. **c,** Cell membrane localization of the TMPRSS2 constructs was monitored by surface immunofluorescence assay. **d,** HeLa cells transfected with the full-length TMPRSS2 or its derivates were exposed to 2 nM TcsH for 3 hours. **c-d,** Red fluorescence (mCherry) marked transfected cells. The scale bar represents 50 µm. Representative images are from one of three independent experiments.

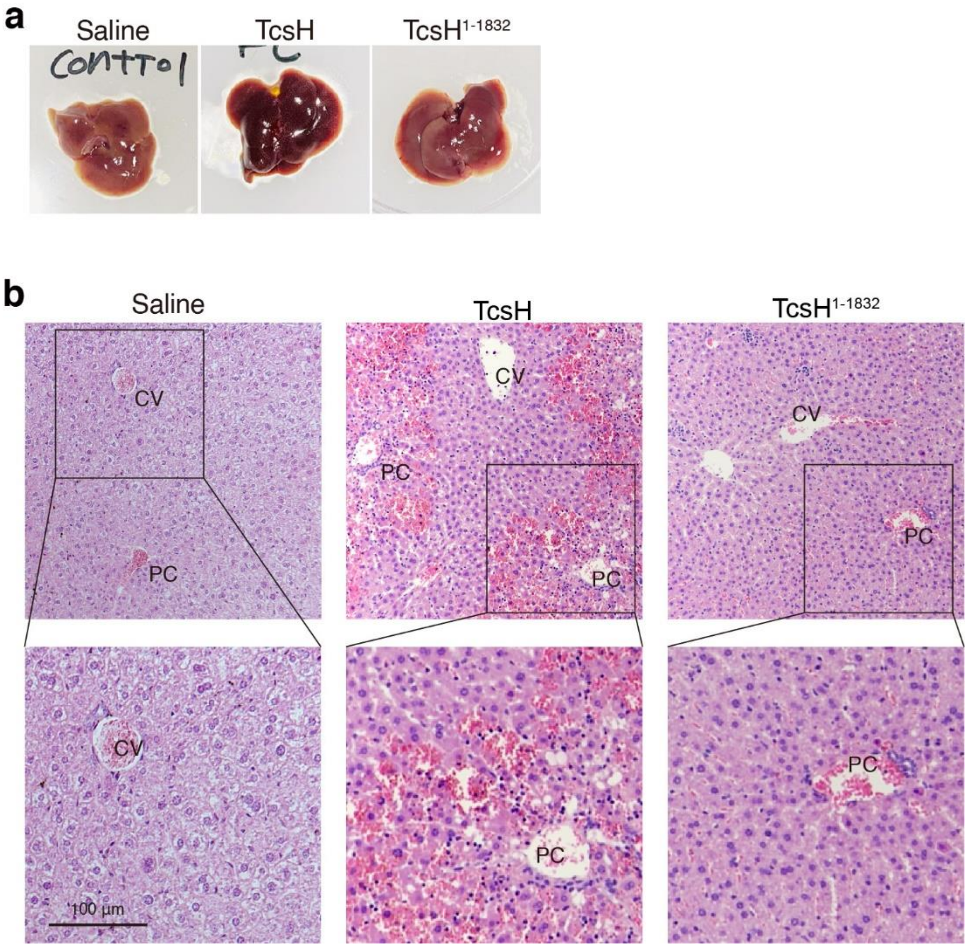


**Supplementary Fig. 9 | The CROPs domain is critical for TcsH toxicity *in vivo*. a,** Mice were intravenously injected with 2 µg/kg TcsH, 2 µg/kg TcsH$^{1-1832}$, or saline. After 8 hours, the mice were euthanized and dissected. The representative pictures of the livers are shown. **b,** Mouse liver tissues in (**a**) were paraffin-embedded, sectioned, and stained by H&E. Representative images are from one of three independent experiments (CV: central vein, PC: portal vein). The scale bar represents 100 µm.

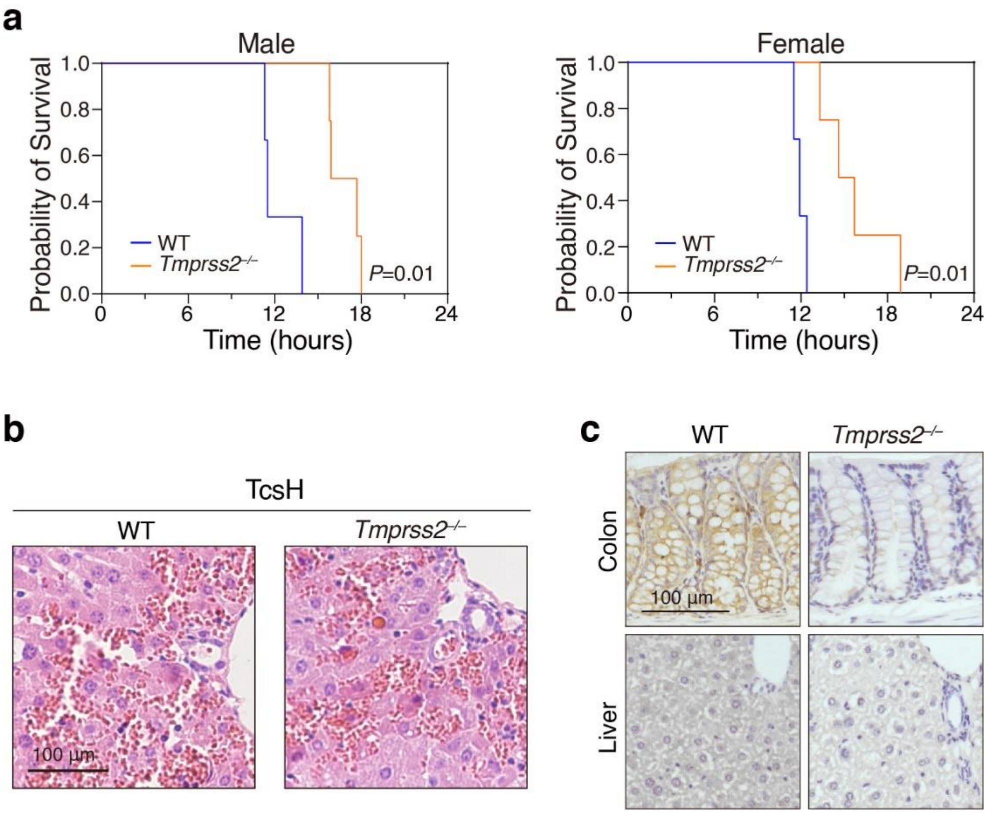


**Supplementary Fig. 10 | Tmprss2 is not responsible for TcsH-induced liver damage *in vivo*. a,** The Kaplan-Meier survival curves of C57BL/6 WT or *Tmprss2*$^{-/-}$ mice after tail vein injection with 2 µg/kg TcsH or saline, separated by the genders. Log-rank (Mantel-Cox) test. **b,** WT or *Tmprss2*$^{-/-}$ mice Mice were intravenously injected with 2 µg/kg TcsH. After 8 hours, the mice were euthanized and their livers were dissected out, paraffin-embedded, sectioned, and stained by H&E. Representative images are from one of three independent experiments. The scale bar represents 100 µm. **c,** IHC staining of tissue sections from the WT or *Tmprss2*$^{-/-}$ mice show that Tmprss2 is mainly expressed in the epithelium of the colon, but not in the liver. The experiments have been repeated independently twice with similar results.

# Full Scans for Supplementary Figure 1 and 8b

## Supplementary Figure 1

MCF-7 WT

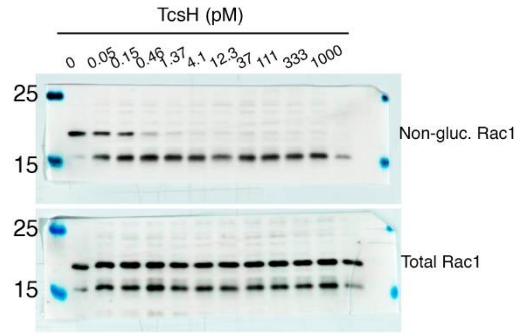


MCF-7 FUT4-/- #25

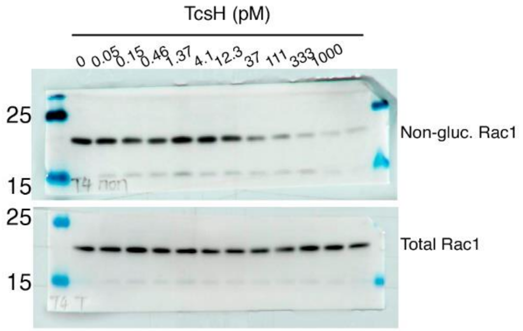


MCF-7 GMDS-/- #13

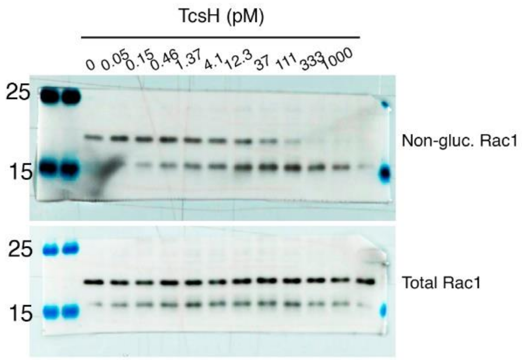


MCF-7 SLC35C1-/- #5

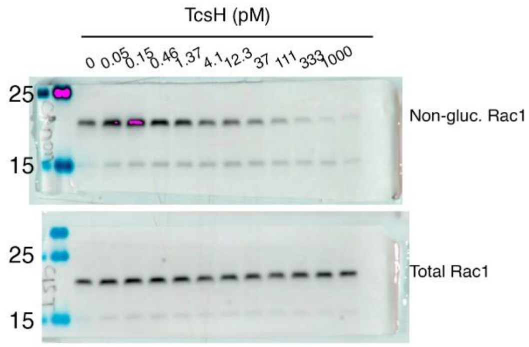


MCF-7 TMPRSS2-/- #3

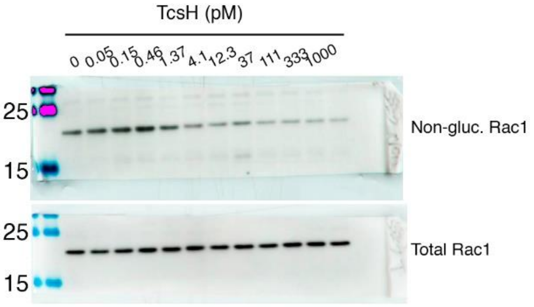


## Supplementary Figure 8b

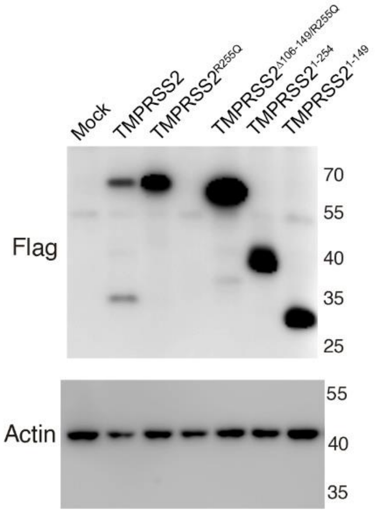

Supplement: Supplementary file 1 — Supplementary Information [file 41467_2022_31994_MOESM1_ESM.pdf]
