# Supplementary material for: Paeniclostridium sordellii hemorrhagic toxin targets TMPRSS2 to induce colonic epithelial lesions
Source: Nat Commun. 2022 Jul 26;13:4331. doi: 10.1038/s41467-022-31994-x (PMC9321280; doi:10.1038/s41467-022-31994-x)
Supplement: Supplementary file 5 — Reporting Summary [file 41467_2022_31994_MOESM5_ESM.pdf]

nature portfolio

Corresponding author(s): Liang Tao

Last updated by author(s): Jun 24, 2022

Reporting Summary

Nature Portfolio wishes to improve the reproducibility of the work that we publish. This form provides structure for consistency and transparency in reporting. For further information on Nature Portfolio policies, see our Editorial Policies and the Editorial Policy Checklist.

Statistics

For all statistical analyses, confirm that the following items are present in the figure legend, table legend, main text, or Methods section.

n/a	Confirmed	
☐	☒	The exact sample size (*n*) for each experimental group/condition, given as a discrete number and unit of measurement
☐	☒	A statement on whether measurements were taken from distinct samples or whether the same sample was measured repeatedly
☐	☒	The statistical test(s) used AND whether they are one- or two-sided<br>*Only common tests should be described solely by name; describe more complex techniques in the Methods section.*
☒	☐	A description of all covariates tested
☒	☐	A description of any assumptions or corrections, such as tests of normality and adjustment for multiple comparisons
☐	☒	A full description of the statistical parameters including central tendency (e.g. means) or other basic estimates (e.g. regression coefficient) AND variation (e.g. standard deviation) or associated estimates of uncertainty (e.g. confidence intervals)
☐	☒	For null hypothesis testing, the test statistic (e.g. *F*, *t*, *r*) with confidence intervals, effect sizes, degrees of freedom and *P* value noted<br>*Give P values as exact values whenever suitable.*
☒	☐	For Bayesian analysis, information on the choice of priors and Markov chain Monte Carlo settings
☒	☐	For hierarchical and complex designs, identification of the appropriate level for tests and full reporting of outcomes
☒	☐	Estimates of effect sizes (e.g. Cohen's *d*, Pearson's *r*), indicating how they were calculated

Our web collection on statistics for biologists contains articles on many of the points above.

Software and code

Policy information about availability of computer code

Data collection	Olympus CellsSens Standard 2.1 and FV31S-SW v2.3.2.169 were used for image data collection.<br>CytoExpert v2.4 (Beckman Coulter) was used for Flow Cytometry data collection.
Data analysis	GraphPad Prism v9.0.0, Octet Data Analysis v12.0.1.2 , CytoExpert v2.4, FlowJo v.10.8.0

For manuscripts utilizing custom algorithms or software that are central to the research but not yet described in published literature, software must be made available to editors and reviewers. We strongly encourage code deposition in a community repository (e.g. GitHub). See the Nature Portfolio guidelines for submitting code & software for further information.

Data

Policy information about availability of data

All manuscripts must include a data availability statement. This statement should provide the following information, where applicable:

- Accession codes, unique identifiers, or web links for publicly available datasets
- A description of any restrictions on data availability
- For clinical datasets or third party data, please ensure that the statement adheres to our policy

The data that support this study are available from the corresponding author upon reasonable request. Source data are provided with this paper.

Field-specific reporting

Please select the one below that is the best fit for your research. If you are not sure, read the appropriate sections before making your selection.

- [x] Life sciences
- [ ] Behavioural & social sciences
- [ ] Ecological, evolutionary & environmental sciences

For a reference copy of the document with all sections, see nature.com/documents/nr-reporting-summary-flat.pdf

Life sciences study design

All studies must disclose on these points even when the disclosure is negative.

Sample size	For all cell rounding experiments: n=6 for each group.<br>For intraperitoneal injection in WT mice with 2 μg/kg TcsH or TcsH1-1832: n=7 mice per group.<br>For toxin challenge assay with 2 μg/kg TcsH: WT mice group (n=6), Tmprss2-/- mice group (n=8).<br>For colon-loop ligation assay: WT+saline (n=6), WT+TcsH (n=6), Tmprss2-/- +TcsH (n=6).<br><br>Sample size was determined based on previous study and knowledge. Each sample size was selected so that a reasonable researcher would conclude that the size is sufficient to draw a statistical conclusion. For in vitro studies, at least two biological replicates were performed. For in vivo studies, 6-8 mice per group is considered sufficient.
Data exclusions	No data exclusions.
Replication	All experiments were replicated at least twice. All attempts at replication are successful.
Randomization	Samples were allocated into experimental groups randomly.
Blinding	The stained tissue sections were scored blinded by two pathologists.<br>For other experiments, blinding was not performed as virtually those data are quantitative and would not easily subject to operator bias.

Reporting for specific materials, systems and methods

We require information from authors about some types of materials, experimental systems and methods used in many studies. Here, indicate whether each material, system or method listed is relevant to your study. If you are not sure if a list item applies to your research, read the appropriate section before selecting a response.

Materials & experimental systems

n/a	Involved in the study	
[ ]	[x]	Antibodies
[ ]	[x]	Eukaryotic cell lines
[x]	[ ]	Palaeontology and archaeology
[ ]	[x]	Animals and other organisms
[x]	[ ]	Human research participants
[x]	[ ]	Clinical data
[x]	[ ]	Dual use research of concern

Methods

n/a	Involved in the study	
[x]	[ ]	ChIP-seq
[ ]	[x]	Flow cytometry
[x]	[ ]	MRI-based neuroimaging

Antibodies

Antibodies used	Mouse monoclonal antibody against non-glucosylated RAC1 (1:1000); BD Biosciences; 610650; Clone 102<br>Mouse monoclonal antibody against total RAC1 (1:1000); Invitrogen; MA1-20580; Clone 23A8<br>Mouse monoclonal antibody against Flag-tag (1:200); GenScript; A01809-100; Clone 5A8E5<br>Rabbit monoclonal antibody against TMPRSS2 (1:2000); Abcam; ab109131; Clone EPR3862<br>Rabbit polyclonal antibody against TMPRSS2 (1:1000); Proteintech; 14437-1-AP<br>Rabbit monoclonal antibody against β-actin (1:5000); Sigma; 078M4809V; Clone AC-15<br>Horseradish peroxidase-labeled goat anti-rabbit IgG (H+L) (1:10000); Vector Labs; PI-1000<br>Horseradish peroxidase-labeled goat anti-mouse IgG (H+L) (1:10000); Vector Labs; PI-2000
Validation	All the antibodies were commercially available and validated by the manufacturers (BD Biosciences, Invitrogen, GenScript, Abcam, Proteintech, Sigma, Vector Labs).<br>Rabbit monoclonal antibody against TMPRSS2 (Abcam, ab109131) were validated to react with human and mouse Tmprss2 and is suitable for WB. (Amarilla et al. Nat Commun 2021, et al.)<br>Rabbit polyclonal antibody against TMPRSS2 (Proteintech, 14437-1-AP) were validated to react with human and mouse Tmprss2 and

is suitable for IHC. (Chadchan et al. Biol Reprod 2021, et al.)
Other antibodies were also validated in our previous experiments and results were published. (Tao et al. Nature 2016; Tao et al. Nat Microbiol 2019; Luo et al. Cell 2022; et al.)

Eukaryotic cell lines

Policy information about cell lines

Cell line source(s)	HeLa (H1, CRL-1958), A549 (CCL-185), U2OS (HTB-96), SH-SY5Y (CRL-2266), HepG2 (HB-8065), 786-O (CRL-1932), MCF-7 (HTB-22), HT-29 (HTB-38), HEK293T (CRL-3216) cells were originally obtained from ATCC. Expi293F (A14527) cells were purchased from ThermoFisher Scientific.
Authentication	HeLa (H1, CRL-1958), HT-29 (HTB-38), and HEK293T (CRL-3216) cells were authenticated via STR profiling (Shanghai Biowing Biotechnology Co. LTD, Shanghai, China). Other cells were not authenticated.
Mycoplasma contamination	They were tested negative for mycoplasma contamination.
Commonly misidentified lines (See ICLAC register)	No commonly misidentified lines were used in this study.

Animals and other organisms

Policy information about studies involving animals; ARRIVE guidelines recommended for reporting animal research

Laboratory animals	C57BL/6 mice were purchased from Laboratory Animal Resources Center at Westlake University (Hangzhou, China). Tmprss2 KO mice were purchased from GemPharmatech (Nanjing, China).<br>Male and female, 6-8 weeks WT and Tmprss2 KO mice were used in this study. Mice were housed in specific-pathogen-free micro-isolator cages with free access to drinking water and food during the experiments.<br>All mice had a 12-hour cycle of light/darkness (7am-7pm), housed at 20-24°C with 40-60% of humidity, and monitored under the care of full-time staff.
Wild animals	No wild animals were used in this study.
Field-collected samples	No field-collected samples were used in this study.
Ethics oversight	All animal procedures reported herein were performed following the institutional guidelines and approved by the Institutional Animal Care and Use Committee at Westlake University (IACUC Protocol #19-010-TL).

Note that full information on the approval of the study protocol must also be provided in the manuscript.

Flow Cytometry

Plots

Confirm that:

- [x] The axis labels state the marker and fluorochrome used (e.g. CD4-FITC).
- [x] The axis scales are clearly visible. Include numbers along axes only for bottom left plot of group (a 'group' is an analysis of identical markers).
- [x] All plots are contour plots with outliers or pseudocolor plots.
- [x] A numerical value for number of cells or percentage (with statistics) is provided.

Methodology

Sample preparation	Cells were trypsinized, washed with PBS, resuspended in carbo-free blocking solution, and incubated for 30 minutes at room temperature. The cells were then washed and stained with 10 μg/mL FITC-LTL or 10 μg/mL FITC-AAL for 20 minutes at 4°C, washed twice with cold blocking solution, and resuspended in PBS for flow cytometry analysis.
Instrument	CytoFLEX LX FC-CEL (Beckman Coulter)
Software	CytoExpert v2.4 (Beckman Coulter)
Cell population abundance	Single cell clones were cultured and used for fucosylation staining.
Gating strategy	FCS/SSC gating strategy was used. The major density of events is captured by this gate. The events with very low FSC and SSC, as well as those with low FSC and high SSC are eliminated. These events represent debris, cell fragments, and pyknotic cells.

- [x] Tick this box to confirm that a figure exemplifying the gating strategy is provided in the Supplementary Information.
